# Clinical Outcomes of High Density Voltage and Fractionation Mapping‐Guided Ablation in Patients With Persistent Atrial Fibrillation

**DOI:** 10.1002/joa3.70398

**Published:** 2026-06-17

**Authors:** Min‐Su Jung, Hyoung‐Seob Park, Jongmin Hwang, Tae‐Wan Chung

**Affiliations:** ^1^ Division of Cardiology, Department of Internal Medicine, Keimyung University School of Medicine Keimyung University Dongsan Hospital Daegu Republic of Korea

## Abstract

**Background:**

Catheter ablation outcomes remain suboptimal in patients with persistent atrial fibrillation (AF), and the optimal strategy beyond pulmonary vein isolation (PVI) has not been established. We evaluated whether a personalized strategy combining high‐density voltage‐guided segmental PVI and fractionation mapping‐guided substrate modification improves clinical outcomes compared to anatomy‐based circumferential PVI.

**Methods:**

This study prospectively enrolled 57 consecutive patients with persistent or long‐standing persistent AF who underwent segmental PVI combined with fractionation mapping‐guided ablation using the EnSite system (2019–2021). Clinical outcomes were compared with a retrospectively identified cohort of 57 patients who underwent anatomy‐based circumferential PVI alone (2017–2021), matched for baseline characteristics. The primary endpoints were 1‐year freedom from AF and AF/atrial tachycardia (AT) recurrence using Kaplan–Meier analysis and log‐rank test. Multivariable Cox proportional hazard regression was performed to adjust for age and left atrial volume.

**Results:**

Kaplan–Meier analysis revealed that the intervention group had a significantly higher 1‐year freedom from AF recurrence compared to the PVI only group (78.9% vs. 59.6%, log‐rank *p* = 0.039). However, 1‐year freedom from any AF/AT recurrence did not reach statistical significance between the two groups (71.9% vs. 56.1%, log‐rank *p* = 0.137). After adjustment for age and left atrial volume, the intervention group showed a trend toward improved AF‐free survival (adjusted hazard ratio [HR] 0.491, 95% CI 0.237–1.016, *p* = 0.055).

**Conclusions:**

In patients with persistent AF, high‐density fractionation mapping–guided ablation in addition to segmental PVI was associated with improved 1‐year freedom from AF. This tailored substrate modification strategy may enhance rhythm control in this challenging population.

## Graphical abstract

High‐density fractionation mapping‐guided ablation combined with voltage‐guided segmental pulmonary vein isolation improved 1‐year freedom from atrial fibrillation recurrence compared with conventional circumferential pulmonary vein isolation in patients with persistent atrial fibrillation, without increasing procedural complications or total ablation time.

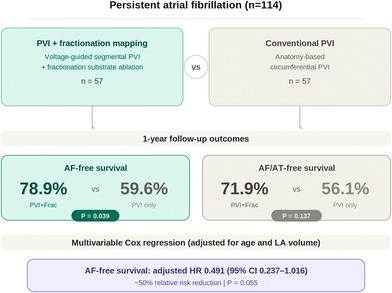

## 1 Introduction

Pulmonary vein isolation (PVI) remains the cornerstone of catheter ablation for atrial fibrillation (AF) [1]. However, its efficacy is limited in patients with persistent AF and long‐standing persistent AF, in whom non‐pulmonary vein substrates are thought to play an important role in arrhythmia maintenance. As a result, various adjunctive ablation strategies beyond PVI have been proposed to improve procedural outcomes in this population. Although substrate modification is a mechanistically appealing approach in AF ablation, procedural success and clinical outcomes have remained inconsistent across studies [2, 3, 4, 5, 6].

Fractionated and continuous atrial electrograms have been considered markers of complex atrial substrate, reflecting areas of slow conduction, wavefront collision, or pivot points of reentrant activity. Such regions may represent sites where multiple wavelets converge or reenter, thereby contributing to the maintenance of AF. Accordingly, complex fractionated atrial electrograms have been proposed as targets for substrate‐based ablation strategies [5].

Recent advances in high‐density electroanatomical mapping have enabled more precise spatiotemporal characterization of these arrhythmogenic substrates. EnSite fractionation mapping using high‐density mapping catheters allows for the identification of regions with repetitive activation patterns and fractionated electrograms independent of AF cycle length, facilitating detailed substrate characterization during AF [7, 8].

Therefore, the purpose of this study was to evaluate the feasibility and clinical outcomes of a personalized ablation strategy combining high‐density voltage‐guided segmental PVI with fractionation mapping‐guided substrate modification, and to compare its preliminary efficacy with the conventional anatomical circumferential PVI approach in patients with persistent AF.

## 2 Methods

### 2.1 Study Population

We prospectively enrolled 57 consecutive patients with persistent and long‐standing persistent AF who underwent catheter ablation at Keimyung University Dongsan Hospital between September 2019 and July 2021. Persistent AF was defined as AF sustained for more than 7 days, and long‐standing persistent AF as continuous AF lasting ≥ 1 year. Patients were eligible if they remained symptomatic despite treatment with at least one class I or III antiarrhythmic drug for more than 6 weeks. Patients were excluded if they were deemed unsuitable for catheter ablation due to a left atrial diameter ≥ 6.0 cm, inability to receive standard therapy such as anticoagulation, prior pulmonary surgery, significant structural heart disease, or a history of prior AF catheter ablation. All patients provided written informed consent, and the study protocol was approved by the Institutional Review Board of Keimyung University Dongsan Hospital. This study was registered at ClinicalTrials.gov (Identifier: NCT03989726).

### 2.2 Study Groups

The intervention group consisted of 57 patients with persistent or long‐standing persistent AF who underwent high‐density voltage and fractionation mapping‐guided AF ablation in addition to segmental PVI. For comparison, a control group of 57 patients with persistent or long‐standing persistent AF who underwent anatomy‐based circumferential PVI was retrospectively identified from the institutional AF ablation database between 2017 and 2021. Control patients were selected using the same inclusion and exclusion criteria to ensure comparable baseline characteristics between the two groups.

### 2.3 Pre‐Procedure Preparation

Antiarrhythmic drugs were discontinued at least five half‐lives before ablation, except amiodarone, to avoid suppression of spontaneous electrical activity and fractionation of the electrograms that can be used to guide ablation. Prior to the procedure, all patients underwent transesophageal echocardiography to screen for left atrial thrombus. Contrast‐enhanced cardiac computed tomography was performed to evaluate left atrial and pulmonary veins anatomy.

Three diagnostic catheters were inserted through the left femoral vein and placed at the right atrium, coronary sinus, and His bundle. Two long sheaths (8.5Fr, Fast‐cath, SL1, St. Jude Medical, Plymouth, MN, USA) were placed in the right atrium through the right femoral vein and inserted to the left atrium using a septal puncture. After septal puncture, intravenous unfractionated heparin was administered to all patients. Activated clotting time (ACT) levels were checked every 10 to 15 min to achieve a target ACT of 300 to 350 s throughout the procedure with intermittent boluses of heparin. A 3.5‐mm irrigated ablation catheter with contact force monitoring, Tacticath Quarts Contact Force Ablation Catheter (St. Jude Medical, St. Paul, MN, USA) and a multielectrode mapping catheter (Inquiry Optima Lasso Catheter or Advisor HD Grid Mapping Catheter, St. Jude Medical, ST. Paul, MN, USA, or other circular mapping catheter) were introduced through the sheaths.

### 2.4 Ablation Procedure

Electroanatomical mapping, including voltage and fractionation mapping, was performed using the Ensite Precision (Abbott Medical, Chicago, USA) system during ongoing AF. Bipolar voltage maps were acquired during ongoing AF using a threshold of < 0.5 mV to define low‐voltage zones. Fractionation mapping was performed using a proprietary algorithm that identifies fractionated electrograms based on the number of deflections per unit time and repetitive activation patterns, regions representing discrete atrial complexes and consistent activation sequences, and does not reflect the cycle length of AF [8]. Unlike conventional complex fractionated atrial electrogram (CFAE) mean mapping, which relies on cycle length–based metrics, this approach allows identification of fractionated regions without dependence on AF cycle length. Map parameters included a width of 5–15 ms, a refractory period of 20–30 ms, and roving sensitivity of 0.1 mV, consistent with previously described methodology [8]. Fractionated signals were collected using a circular mapping catheter or a multipolar mapping catheter (Advisor HD Grid).

First, a voltage‐map guided segmental PVI was performed, which prioritized the isolation of PVs by specifically targeting preserved high‐voltage regions (> 0.5 mV) within the antral areas. Unlike routine circumferential ablation, this approach focused on eliminating identified conduction gaps via high‐density mapping to achieve complete electrical isolation while minimizing unnecessary tissue injury. If electrical isolation was not achieved, additional segmental ablation guided by a circular mapping catheter was performed. Radiofrequency energy was delivered at target sites for 15–30 s with a target contact force of 5–20 g (typically 10–20 g for anterior wall lesions and 5–15 g for posterior wall lesions). The target lesion size index (LSI) was ≥ 5.0 for anterior lesions and ≥ 4.0 for posterior lesions, with real‐time monitoring of local electrogram attenuation to confirm adequate tissue modification. Successful PVI was confirmed by demonstration of entrance and/or exit block.

**FIGURE 1:**
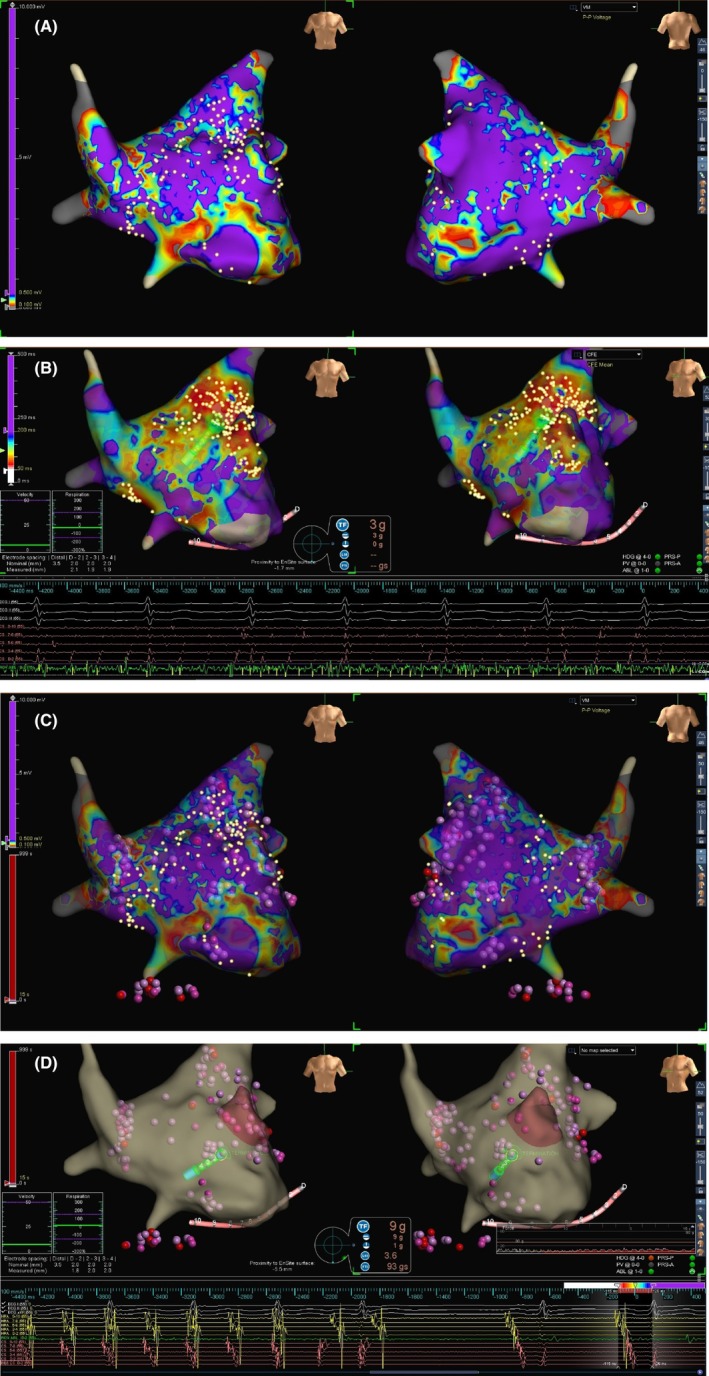
Representative case of fractionation mapping‐guided ablation in a patient with persistent atrial fibrillation (AF). (A) Baseline high‐density maps: combined high‐density voltage and fractionation maps of the left atrium (LA) in anteroposterior (AP) and posteroanterior (PA) views. The maps provide a detailed characterization of the atrial substrate; yellow dots represent the identified fractionation targets. (B) Intracardiac electrogram during fractionation ablation: representative electrogram recordings obtained during radiofrequency ablation of a fractionation site (yellow dot) using a TactiCath Quartz Contact Force Ablation Catheter. The fractionation electrogram demonstrates repetitive activation patterns with multiple deflections independent of AF cycle length. (C) Integrated ablation lesion sets: A comprehensive map of all radiofrequency ablation sites (pink/purple dots) delivered during the procedure. The lesion sets include segmental pulmonary vein isolation (PVI), fractionation mapping‐guided ablation (targeting the sites identified in Panel A), cavotricuspid isthmus (CTI) linear ablation, and subsequent focal ablation for organized atrial tachycardia (AT). (D) Tachycardia organization and termination: following PVI and fractionation mapping‐guided ablation, AF organized into an atrial flutter. Subsequent CTI linear ablation resulted in a significant prolongation of cycle length and a change in the activation sequence. Activation mapping then identified a localized reentrant AT involving the anterior LA wall (base of the left atrial appendage). Targeted ablation at this critical isthmus led to the immediate termination of the AT to sinus rhythm, as demonstrated in the intracardiac electrogram recording.

If AF persisted after PVI, additional fractionation mapping‐guided ablation was performed. The fractionation area within low voltage zones, which were defined as an area with bipolar peak‐to‐peak voltage amplitudes < 0.5 mV, was targeted. If AF organized into atrial flutter or atrial tachycardia during ablation, detailed activation mapping was performed to identify the underlying mechanism, followed by targeted ablation of the responsible circuit. If AF persisted even after fractionation mapping‐guided ablation, electrical cardioversion was conducted to restore sinus rhythm. Induction test was then performed using adenosine injection under isoproterenol infusion. Cavo‐tricuspid isthmus (CTI) linear ablation was performed when typical atrial flutter was documented clinically or induced during the electrophysiologic study. A representative example of fractionation mapping‐guided ablation using a high‐density electroanatomical mapping system is shown in Figure 1. Zero‐ or near‐zero fluoroscopy strategies were used at the operator's discretion, particularly during procedures guided by high‐density electroanatomical mapping.

### 2.5 Outcomes

The primary endpoint was freedom from any atrial tachyarrhythmia recurrence lasting longer than 30 s during the 1‐year follow‐up period after a single ablation procedure, with or without the use of anti‐arrhythmic medications, following a 3‐month blanking period.

The secondary endpoints included total procedure time, fluoroscopy time, ablation time, redo ablation rate, and maintenance of sinus rhythm after 1 year.

### 2.6 Follow‐Up

Patients underwent 12‐lead electrocardiography and 24‐h Holter monitoring immediately after the procedure and at 3, 6, and 12 months. Recurrence was defined as documentation of AF or atrial tachycardia (AT) lasting longer than 30 s after the 3‐month blanking period.

### 2.7 Statistical Analysis

Categorical variables were compared using Fisher's exact test or chi‐squared test, as appropriate. Continuous variables were compared using the student's *t*‐test or the Mann–Whitney *U*‐test according to data distribution. Kaplan–Meier survival curves were constructed to assess time to first recurrence of atrial arrhythmia, and comparisons between groups were performed using the log‐rank (Mantel–Cox) test. To assess the independent association between fractionation mapping‐guided ablation and clinical outcomes after adjusting for potential confounders, multivariable Cox proportional hazard models were constructed. Covariates were selected a priori based on clinical relevance and included age and left atrial volume. The proportional hazards assumption was verified using log‐minus‐log plots. Propensity score matching was considered but not performed because the baseline characteristics were already well‐balanced between groups, the sample size was small, and matching would have further reduced the analyzable cohort. Post hoc statistical power was calculated using G*Power software (version 3.1.9.7) for the composite AF/AT‐free survival endpoint. A two‐sided *p* < 0.05 was considered statistically significant.

Because this study consisted of a prospectively enrolled intervention cohort and a retrospectively selected control cohort, no formal sample size calculation was performed. The sample size was determined by the total number of consecutive patients who underwent fractionation mapping–guided ablation during the predefined study period. All analyses were conducted on an as‐treated basis. The data analyses were performed with IBM SPSS Statistics version 23 software (IBM Corp., Armonk, NY, USA).

## 3 Results

### 3.1 Patient Characteristics

A total of 114 patients were included in the analysis, with 57 patients assigned to each of the PVI plus fractionation mapping‐guided ablation group and the PVI only group. The mean age of the overall cohort was 57.7 ± 10.0 years, and 81 (71.1%) of them were male. The mean left atrial dimension of total patients was 4.6 ± 0.5 cm and the mean duration of AF was 40.4 ± 47.0 months. Baseline clinical and echocardiographic characteristics were well balanced between the two groups. Detailed baseline characteristics are summarized in Table 1.

**TABLE 1:** Baseline characteristics of patients.

	Total (*n* = 114)	PVI + Fractionation (*n* = 57)	PVI (*n* = 57)	*p*
Age, years	57.7 ± 10.0	59.5 ± 8.9	55.9 ± 10.8	0.053
Female, *n* (%)	33 (28.9)	17 (29.8)	16 (28.1)	0.836
Male, *n* (%)	81 (71.1)	40 (70.2)	41 (71.9)	
Height, cm	166.1 ± 8.7	165.1 ± 8.7	167.2 ± 8.6	0.212
Weight, kg	72.4 ± 13.0	69.8 ± 11.9	75.0 ± 13.7	0.032
BSA, m^2^	1.8 ± 0.2	1.8 ± 0.2	1.9 ± 0.2	0.041
BMI, kg/m^2^	26.1 ± 3.5	25.5 ± 3.1	26.7 ± 3.9	0.055
Risk factor				
HF, *n* (%)	18 (15.8)	11 (19.3)	7 (12.3)	0.304
HTN, *n* (%)	51 (44.7)	27 (47.4)	24 (42.1)	0.572
DM, *n* (%)	23 (20.2)	11 (19.3)	12 (21.1)	0.815
History of a CVA, *n* (%)	11 (9.6)	6 (10.5)	5 (8.8)	0.751
Peripheral vascular disease, *n* (%)	7 (6.1)	5 (8.8)	2 (3.5)	0.438
CH $A_{2}$ D $S_{2}$ ‐Vasc Score	1.6 ± 1.4	1.8 ± 1.5	1.5 ± 1.3	0.303
AF duration, months	40.4 ± 47.0	41.4 ± 53.9	39.3 ± 39.3	0.486
Echocardiography				
EF, %	55.1 ± 10.8	53.1 ± 11.7	57.1 ± 9.6	0.089
LAD, cm	4.6 ± 0.5	4.6 ± 0.5	4.7 ± 0.6	0.520
LAV, mL	99.5 ± 22.0	103.7 ± 22.9	95.3 ± 20.5	0.104

### 3.2 Procedural Results

In the PVI plus fractionation mapping‐guided ablation group, AF terminated to sinus rhythm or AT in 18 patients (31.6%) during the procedure, including direct conversion to sinus rhythm in 10 patients and conversion to AT in 8 patients. In the PVI only group, AF terminated to sinus rhythm in 8 patients (14.0%) during ablation. Of the 18 patients with procedural AF termination, 10 experienced termination during segmental PVI (sinus rhythm conversion in 7, AT organization in 3) and 8 during fractionation mapping‐guided ablation (sinus rhythm conversion in 3, AT organization in 5).

Additional CTI linear ablation for CTI dependent macroreentrant atrial flutter was performed more frequently in the PVI plus fractionation mapping‐guided ablation group than in the PVI only group (44 patients [77.2%] vs. 8 patients [14.0%]). In the PVI plus fractionation mapping‐guided ablation group, additional right atrium ablation was performed in 16 patients (28%).

Although the total procedure time was significantly longer in the PVI plus fractionation mapping‐guided ablation group compared with the PVI only group (154.8 ± 31.7 vs. 137.9 ± 43.6 min, *p* = 0.008), there was no significant difference in total radiofrequency ablation time between the two groups (32.7 ± 9.5 vs. 34.8 ± 13.4 min, *p* = 0.756). In contrast, fluoroscopy time was significantly shorter in the fractionation mapping group (8.0 ± 11.2 vs. 17.3 ± 10.2 min, *p* < 0.001). No major procedure‐related complications occurred.

### 3.3 Clinical Follow‐Up Results

**FIGURE 2:**
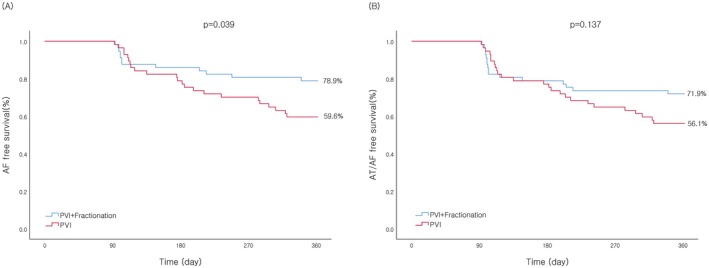
Comparison of the Kaplan–Meier curves for the 1‐year atrial tachyarrhythmias‐free survival after ablation between the two groups. (A) The pulmonary vein isolation (PVI) plus fractionation mapping‐guided ablation group had a higher 1‐year atrial fibrillation‐free survival than the PVI only group. (B) There was no significant difference in the 1‐year atrial fibrillation/atrial tachycardia free survival between the two groups. *p* values were derived from the log‐rank (Mantel–Cox) test.

During the 1‐year follow‐up period, freedom from AF was higher in the PVI plus fractionation mapping‐guided ablation group. Kaplan–Meier analysis demonstrated a significantly higher 1‐year AF‐free survival in the fractionation mapping group compared with the PVI only group (78.9% vs. 59.6%, log‐rank *p* = 0.039; Figure 2A). However, freedom from any atrial tachyarrhythmia (AF/AT) did not differ significantly between the two groups on Kaplan–Meier analysis (71.9% vs. 56.1%, log‐rank *p* = 0.137; Figure 2B). During the follow‐up period, redo ablation procedures were performed in 8 patients in the fractionation mapping group and in 7 patients in the PVI only group. After redo procedures, maintenance of sinus rhythm at 1 year was achieved in 40 patients (70.2%) in the fractionation mapping group and in 28 patients (49.1%) in the PVI only group (*p* = 0.022).

Multivariable Cox proportional hazard regression was performed to evaluate the independent effect of fractionation mapping‐guided ablation after adjusting for age and left atrial volume. The intervention group showed a trend toward improved AF‐free survival (adjusted HR 0.491, 95% CI 0.237–1.016, *p* = 0.055), with a clinically meaningful approximately 50% relative risk reduction. For the composite AF/AT‐free survival endpoint, the adjusted HR was 0.626 (95% CI 0.326–1.204, *p* = 0.160), consistent in direction with the primary endpoint.

No late procedure‐related complications were observed during follow‐up. One patient in the fractionation mapping group required permanent pacemaker implantation due to sinus node dysfunction with junctional escape rhythm following the procedure.

## 4 Discussion

In the present study, we found that segmental PVI combined with fractionation mapping‐guided ablation was associated with improved 1‐year freedom from AF in patients with persistent and long‐standing persistent AF. However, there was no significant difference in overall atrial tachyarrhythmia‐free survival between the two groups.

Previous studies have consistently reported a high recurrence rate after catheter ablation in patients with persistent and long‐standing persistent AF [9, 10]. In an effort to improve outcomes, various ablation targets beyond PVI have been investigated including the left atrial appendage, posterior wall, ligament of Marshall, or areas of atrial scar [11, 12, 13, 14, 15]. However, the clinical outcomes of these approaches have been inconsistent, and no single strategy has demonstrated universal efficacy across all patient populations [10, 16]. This variability likely reflects the heterogeneous mechanisms underlying AF among individuals. Therefore, efforts have been made to develop more personalized adjunctive ablation strategies tailored to individual atrial substrates. These approaches include targeting areas of atrial fibrosis or low‐voltage regions, as well as presumed electrical drivers of AF such as CFAE [11, 17, 18, 19, 20]. However, the heterogeneous nature of atrial remodeling in persistent AF [21] has limited the reproducibility and overall efficacy of these strategies.

Recent advances in high‐density electroanatomical mapping systems have enabled more detailed characterization of atrial substrate heterogeneity. Fractionation mapping allows visualization of regions with clustered fragmented electrograms, facilitating identification of areas that may represent potential arrhythmogenic substrates [8]. By enabling more precise localization of fragmented regions, fractionation mapping may assist in tailoring substrate modification beyond empirical lesion sets. This approach allows for a more tailored ablation strategy by targeting patient‐specific electrophysiological substrates rather than applying uniform lesion sets. In the present study, we therefore aimed to evaluate whether a fractionation mapping‐guided ablation strategy, applied in addition to segmental PVI, could improve ablation outcomes in patients with persistent and long‐standing persistent AF by more accurately targeting individualized arrhythmogenic substrates.

In addition to substrate modification, the use of voltage‐guided segmental PVI in the fractionation mapping‐guided group represents a more refined approach compared to conventional circumferential ablation [22]. Instead of applying continuous lesion set around the PV antrum, this strategy focuses on identifying and eliminating specific conduction gaps within high‐voltage regions. By targeting only the functional electrical connections, we were able to achieve complete PV isolation while minimizing unnecessary myocardial injury.

Notably, in the fractionation mapping‐guided group, AF was converted to either AT or sinus rhythm during the procedure in 18 patients (31.6%), whereas such procedural termination or organization occurred in only 8 patients (14.0%) in the PVI only group. Of these 18 patients, AT organization occurred more frequently during fractionation ablation (5/8) than during PVI (3/8), consistent with the mechanistic expectation that substrate modification eliminates disorganized AF drivers and unmasks organized reentrant circuits. When AF organized into AT during AF ablation, we performed detailed activation mapping to identify the underlying circuit and targeted these sites for further ablation. In fact, the maintenance of sinus rhythm at 1 year—reflecting the final clinical status after accounting for successful redo procedures—was significantly higher in the intervention group (70.2% vs. 49.1%, *p* = 0.022). These data suggest that the systematic identification and ablation of organized tachyarrhythmias, coupled with precise substrate modification, may contribute to achieving a more stable rhythm status at the 1‐year follow‐up, even when transient recurrences occur. Indeed, previous studies have reported that both AF termination to sinus rhythm and its organization into AT during ablation are associated with favorable clinical outcomes and significantly higher success rates of long‐term rhythm restoration compared to patients with persistent AF recurrence [23, 24, 25, 26].

The current study demonstrated that while fractionation mapping‐guided ablation significantly improved 1‐year AF‐free survival (78.9% vs. 59.6%, *p* = 0.039), the overall freedom from any atrial tachyarrhythmia did not reach statistical significance (71.9% vs. 56.1%, *p* = 0.137). This discrepancy is largely explained by the modest numerical increase in recurrent AT within the intervention group (8.8% vs. 7.0%, *p* = 0.728), which partially offset the clinical gain achieved in AF suppression. It is important to note that the overall arrhythmia burden and the necessity for redo procedures remained comparable between the groups (14.0% vs. 12.3%, *p* = 0.782), suggesting that the more extensive substrate modification targeting fractionation and low‐voltage zones did not result in a clinically meaningful increase in recurrent atrial arrhythmias. While this study was not powered to detect subtle differences in AT mechanisms, the significantly higher rate of sinus rhythm maintenance after redo procedures in the intervention group (70.2% vs. 49.1%, *p* = 0.022) suggests that the substrates targeted during the initial procedure may contribute to more favorable long‐term rhythm control.

The choice of AF‐free survival as a co‐primary endpoint deserves further consideration. AF represents the predominant arrhythmia mechanism in persistent AF and is the primary target of substrate modification, and the ability to eliminate AF reflects the effectiveness of the ablation strategy against the underlying pathological substrate. Furthermore, the occurrence of organized AT following substrate ablation may reflect successful elimination of disorganized AF substrate rather than treatment failure, as AF organization into AT during ablation has been associated with favorable long‐term outcomes in previous studies [23, 26]. Recurrent AT is also typically amenable to targeted catheter ablation with high success rates, whereas recurrent AF often represents persistent or recurrent substrate pathology that is more difficult to treat.

To address potential selection bias inherent in the non‐randomized design, multivariable Cox regression was performed adjusting for age and left atrial volume. The intervention group showed a trend toward improved AF‐free survival with an adjusted HR of 0.491 (95% CI 0.237–1.016, *p* = 0.055), corresponding to an approximately 50% relative risk reduction. Although this did not reach conventional statistical significance, the magnitude and direction of the effect were consistent with the unadjusted analysis, supporting the independent association between fractionation mapping‐guided ablation and improved AF outcomes. The borderline statistical significance after adjustment likely reflects the limited statistical power inherent in the small sample size.

The higher rate of CTI linear ablation in the intervention group (77.2% vs. 14.0%) likely reflects the more comprehensive electrophysiologic evaluation protocol in the intervention group, including more aggressive arrhythmia induction testing, which may have led to more frequent identification of CTI‐dependent atrial flutter. However, given the substantial difference in CTI ablation rates between groups, the potential influence of these additional procedures on overall outcomes cannot be entirely excluded, and this remains a limitation of the current study.

An interesting finding in our study was that despite the additional fractionation mapping and substrate modification in the intervention group, total procedure and ablation times were not significantly different compared to the conventional PVI group. Several factors may explain this observation. First, the intervention group underwent voltage‐guided segmental PVI rather than wide circumferential ablation. This segmental approach targets high‐voltage gaps (> 0.5 mV) rather than creating continuous circumferential lesions, potentially requiring fewer radiofrequency applications despite additional substrate targets [22]. Second, fractionation mapping allowed targeted substrate modification at discrete high‐fractionation regions rather than empirical linear lesion sets. This focused strategy may be more time‐efficient than creating extensive linear lesions while still addressing relevant substrate. Third, the intervention procedures (2019–2021) benefited from accumulated institutional experience with high‐density mapping workflows, which may have improved procedural efficiency over time.

Furthermore, despite the more extensive ablation strategy in the intervention group, the fluoroscopy time was significantly shorter compared with the control group. This finding is particularly notable and likely reflects the routine integration of high‐density electroanatomical mapping and structured zero‐ or near‐zero fluoroscopy workflow. By enabling precise catheter navigation and lesion delivery without constant fluoroscopic guidance, this approach not only improves procedural efficiency but also enhances safety for both patients and medical staff.

### 4.1 Limitations

This study has several limitations. First, this was a single‐center study with a relatively small sample size, which may limit generalizability of our findings. Importantly, post hoc power analysis estimated only 41.9% statistical power to detect the observed 15.8% absolute difference in the composite endpoint with the current sample size, suggesting that the non‐significant result may partly reflect insufficient sample size to detect a true difference; however, a genuinely limited treatment effect on the composite endpoint cannot be excluded. To achieve 80% power for this effect size, approximately 144 patients per group would be required. Additionally, although the unadjusted log‐rank analysis demonstrated significantly improved AF‐free survival (*p* = 0.039), multivariable Cox regression showed only a borderline significant trend (adjusted HR 0.491, 95% CI 0.237–1.016, *p* = 0.055), further reflecting the limited power for adjusted analysis. These findings underscore the need for larger, multicenter randomized trials to confirm the efficacy of fractionation mapping‐guided ablation in persistent AF.

Second, the non‐randomized design with retrospective control group selection introduces significant potential for bias. Although multivariable Cox regression was performed to adjust for measured confounders, this approach cannot fully account for residual confounding due to unmeasured variables. The temporal difference in enrollment periods (control: 2017–2021; intervention: 2019–2021) means that practice patterns, institutional protocols, and operator experience may have evolved between groups, potentially confounding the comparison. These inherent limitations of the study design should be considered when interpreting the results, and a prospective randomized trial would be required to establish a causal relationship between fractionation mapping‐guided ablation and improved outcomes. Third, all procedures were performed by experienced operators at a single high‐volume tertiary center, which may limit generalizability to other practice settings. Fourth, while the 1‐year follow‐up results are encouraging, longer‐term data are necessary to evaluate the late durability of fractionation mapping‐guided substrate modification.

## 5 Conclusion

In conclusion, this comparative study demonstrates that high‐density voltage and fractionation mapping‐guided ablation in addition to segmental PVI was associated with improved 1‐year freedom from AF compared with conventional PVI alone in patients with persistent AF, without an increase in procedural complications. These findings suggest that a tailored substrate modification strategy guided by high‐density electroanatomical mapping may enhance rhythm control in this challenging patient population. However, these results warrant validation in a prospective randomized controlled trial before definitive conclusions can be drawn about the superiority of this approach.

## Funding

This work was supported by a research grant from Abbott Inc.

## Ethics Statement

This study was approved by the Institutional Review Board of Keimyung University Dongsan Hospital (Approval No. 2018‐12‐020). Informed consent was obtained. The requirement for informed consent was waived for the retrospective cohort by the Institutional Review Board.

## Conflicts of Interest

Dr. Hyoung‐Seob Park has received research grants from Abbott Inc. The other authors declare no conflicts of interest.

## Data Availability

The data that support the findings of this study are available from the corresponding author upon reasonable request.

## References

[1] S. Tzeis , E. P. Gerstenfeld , J. Kalman , et al., “European Heart Rhythm Association/Heart Rhythm Society/Asia Pacific Heart Rhythm Society/Latin American Heart Rhythm Society Expert Consensus Statement on Catheter and Surgical Ablation of Atrial Fibrillation,” Europace 26, no. 4 (2024): euae043. 38587017 10.1093/europace/euae043 PMC11000153

[2] A. G. Brooks , M. K. Stiles , J. Laborderie , et al., “Outcomes of Long‐Standing Persistent Atrial Fibrillation Ablation: A Systematic Review,” Heart Rhythm 7, no. 6 (2010): 835–846. 20206320 10.1016/j.hrthm.2010.01.017

[3] M. Haïssaguerre , M. É. L. È. Z. E. Hocini , P. R. A. S. H. A. N. T. H. A. N. Sanders , et al., “Catheter Ablation of Long‐Lasting Persistent Atrial Fibrillation: Clinical Outcome and Mechanisms of Subsequent Arrhythmias,” Journal of Cardiovascular Electrophysiology 16, no. 11 (2005): 1138–1147. 16302893 10.1111/j.1540-8167.2005.00308.x

[4] H. Kottkamp , J. Berg , R. Bender , A. Rieger , and D. Schreiber , “Box Isolation of Fibrotic Areas (BIFA): A Patient‐Tailored Substrate Modification Approach for Ablation of Atrial Fibrillation,” Journal of Cardiovascular Electrophysiology 27, no. 1 (2016): 22–30. 26511713 10.1111/jce.12870

[5] K. Nademanee , J. McKenzie , E. Kosar , et al., “A New Approach for Catheter Ablation of Atrial Fibrillation: Mapping of the Electrophysiologic Substrate,” Journal of the American College of Cardiology 43, no. 11 (2004): 2044–2053. 15172410 10.1016/j.jacc.2003.12.054

[6] A. Verma , C. Y. Jiang , T. R. Betts , et al., “Approaches to Catheter Ablation for Persistent Atrial Fibrillation,” New England Journal of Medicine 372, no. 19 (2015): 1812–1822. 25946280 10.1056/NEJMoa1408288

[7] T. Aksu , T. E. Guler , S. Bozyel , and K. Yalin , “Usage of a New Mapping Algorithm to Detect Possible Critical Substrate for Continuity of Atrial Fibrillation: Fractionation Mapping in Preliminary Experience,” Journal of Interventional Cardiac Electrophysiology 58, no. 1 (2020): 29–34. 31984467 10.1007/s10840-019-00693-x

[8] T. Aksu , T. E. Guler , S. Bozyel , K. Yalin , D. Lakkireddy , and R. Gopinathannair , “Initial Experience With Fractionation Mapping‐Guided Ablation Strategy in Patients With Long‐Standing Persistent Atrial Fibrillation,” Journal of Interventional Cardiac Electrophysiology 61, no. 2 (2021): 405–413. 32712899 10.1007/s10840-020-00834-7

[9] R. R. Tilz , A. Rillig , A. M. Thum , et al., “Catheter Ablation of Long‐Standing Persistent Atrial Fibrillation: 5‐Year Outcomes of the Hamburg Sequential Ablation Strategy,” Journal of the American College of Cardiology 60, no. 19 (2012): 1921–1929. 23062545 10.1016/j.jacc.2012.04.060

[10] J. A. Joglar , M. K. Chung , A. L. Armbruster , et al., “2023 ACC/AHA/ACCP/HRS Guideline for the Diagnosis and Management of Atrial Fibrillation: A Report of the American College of Cardiology/American Heart Association Joint Committee on Clinical Practice Guidelines,” Circulation 149, no. 1 (2024): e1. 38033089 10.1161/CIR.0000000000001193 PMC11095842

[11] N. F. Marrouche , O. Wazni , C. McGann , et al., “Effect of MRI‐Guided Fibrosis Ablation vs Conventional Catheter Ablation on Atrial Arrhythmia Recurrence in Patients With Persistent Atrial Fibrillation: The DECAAF II Randomized Clinical Trial,” JAMA 327, no. 23 (2022): 2296–2305. 35727277 10.1001/jama.2022.8831 PMC9214588

[12] C. Gianni , S. Mohanty , L. di Biase , et al., “Acute and Early Outcomes of Focal Impulse and Rotor Modulation (FIRM)‐Guided Rotors‐Only Ablation in Patients With Nonparoxysmal Atrial Fibrillation,” Heart Rhythm 13, no. 4 (2016): 830–835. 26706193 10.1016/j.hrthm.2015.12.028

[13] L. Di Biase , J. D. Burkhardt , P. Mohanty , et al., “Left Atrial Appendage Isolation in Patients With Longstanding Persistent AF Undergoing Catheter Ablation: BELIEF Trial,” Journal of the American College of Cardiology 68, no. 18 (2016): 1929–1940. 27788847 10.1016/j.jacc.2016.07.770

[14] P. M. Kistler , D. Chieng , H. Sugumar , et al., “Effect of Catheter Ablation Using Pulmonary Vein Isolation With vs Without Posterior Left Atrial Wall Isolation on Atrial Arrhythmia Recurrence in Patients With Persistent Atrial Fibrillation: The CAPLA Randomized Clinical Trial,” JAMA 329, no. 2 (2023): 127–135. 36625809 10.1001/jama.2022.23722 PMC9856612

[15] M. Valderrábano , L. E. Peterson , V. Swarup , et al., “Effect of Catheter Ablation With Vein of Marshall Ethanol Infusion vs Catheter Ablation Alone on Persistent Atrial Fibrillation: The VENUS Randomized Clinical Trial,” JAMA 324, no. 16 (2020): 1620–1628. 33107945 10.1001/jama.2020.16195 PMC7592031

[16] A. Sau , S. Kapadia , S. al‐Aidarous , et al., “Temporal Trends and Lesion Sets for Persistent Atrial Fibrillation Ablation: A Meta‐Analysis With Trial Sequential Analysis and Meta‐Regression,” Circulation. Arrhythmia and Electrophysiology 16, no. 9 (2023): e011861. 37589197 10.1161/CIRCEP.123.011861 PMC10510845

[17] M. Efremidis , K. Vlachos , K. P. Letsas , et al., “Targeted Ablation of Specific Electrogram Patterns in Low‐Voltage Areas After Pulmonary Vein Antral Isolation in Persistent Atrial Fibrillation: Termination to an Organized Rhythm Reduces Atrial Fibrillation Recurrence,” Journal of Cardiovascular Electrophysiology 30, no. 1 (2019): 47–57. 30288830 10.1111/jce.13763

[18] I. Sim , M. Bishop , M. O'Neill , and S. E. Williams , “Left Atrial Voltage Mapping: Defining and Targeting the Atrial Fibrillation Substrate,” Journal of Interventional Cardiac Electrophysiology 56, no. 3 (2019): 213–227. 31076965 10.1007/s10840-019-00537-8 PMC6900285

[19] K. Nademanee , E. Lockwood , N. Oketani , and B. Gidney , “Catheter Ablation of Atrial Fibrillation Guided by Complex Fractionated Atrial Electrogram Mapping of Atrial Fibrillation Substrate,” Journal of Cardiology 55, no. 1 (2010): 1–12. 20122543 10.1016/j.jjcc.2009.11.002

[20] J. Seitz , C. Bars , G. Théodore , et al., “AF Ablation Guided by Spatiotemporal Electrogram Dispersion Without Pulmonary Vein Isolation: A Wholly Patient‐Tailored Approach,” Journal of the American College of Cardiology 69, no. 3 (2017): 303–321. 28104073 10.1016/j.jacc.2016.10.065 PMC5568427

[21] A. W. Teh , P. M. Kistler , G. Lee , et al., “Electroanatomic Remodeling of the Left Atrium in Paroxysmal and Persistent Atrial Fibrillation Patients Without Structural Heart Disease,” Journal of Cardiovascular Electrophysiology 23, no. 3 (2012): 232–238. 21955090 10.1111/j.1540-8167.2011.02178.x

[22] H. Oral , B. P. Knight , M. Özaydın , et al., “Segmental Ostial Ablation to Isolate the Pulmonary Veins During Atrial Fibrillation: Feasibility and Mechanistic Insights,” Circulation 106, no. 10 (2002): 1256–1262. 12208802 10.1161/01.cir.0000027821.55835.00

[23] S. Ammar , G. Hessling , T. Reents , et al., “Arrhythmia Type After Persistent Atrial Fibrillation Ablation Predicts Success of the Repeat Procedure,” Circulation. Arrhythmia and Electrophysiology 4, no. 5 (2011): 609–614. 21856772 10.1161/CIRCEP.111.963256

[24] Y. Choi , S. Kim , J. Y. Baek , et al., “Acute and Long‐Term Outcome of Redo Catheter Ablation for Recurrent Atrial Tachycardia and Recurrent Atrial Fibrillation in Patients With Prior Atrial Fibrillation Ablation,” Journal of Interventional Cardiac Electrophysiology 61, no. 2 (2021): 227–234. 32556924 10.1007/s10840-020-00795-x

[25] D. Schreiber , T. Rostock , M. Fröhlich , et al., “Five‐Year Follow‐Up After Catheter Ablation of Persistent Atrial Fibrillation Using the Stepwise Approach and Prognostic Factors for Success,” Circulation. Arrhythmia and Electrophysiology 8, no. 2 (2015): 308–317. 25744570 10.1161/CIRCEP.114.001672

[26] M. D. O'Neill , M. Wright , S. Knecht , et al., “Long‐Term Follow‐Up of Persistent Atrial Fibrillation Ablation Using Termination as a Procedural Endpoint,” European Heart Journal 30, no. 9 (2009): 1105–1112. 19270341 10.1093/eurheartj/ehp063

